# The Diatom *Odontella aurita* Modulates Melanogenesis in *B16-F0* Cell Line

**DOI:** 10.3390/antiox14121402

**Published:** 2025-11-25

**Authors:** Clementina Sansone, Luigi Pistelli, Debora Paris, Annabella Tramice, Annalaura Iodice, Christophe Brunet

**Affiliations:** 1 Stazione Zoologica Anton Dohrn, Villa Comunale, 80121 Naples, Italy; luigi.pistelli@szn.it (L.P.); christophe.brunet@szn.it (C.B.); 2 Institute of Biomolecular Chemistry, National Research Council, 80078 Pozzuoli, Italy; debora.paris@cnr.it (D.P.); annabella.tramice@cnr.it (A.T.); annalauraiodice@cnr.it (A.I.)

**Keywords:** diatom, tyrosinase, signaling pathway, melanin, metabolomics

## Abstract

Melanin, a pigment synthesized by melanocytes, serves as the primary defense against UV-induced skin damage due to its potent antioxidant properties. There is increasing interest in natural substances capable of modulating the melanogenic pathway, particularly in hypopigmentation disorders. This study investigated the effect of methanolic extracts from the diatom *Odontella aurita*—authorized as a food supplement in the EU—on melanogenesis in the B16-F0 murine melanoma cell line. The research evaluated melanin content, tyrosinase activity, and the expression of melanogenesis-related genes and proteins at defined time points. Metabolomic and biochemical analyses were performed to characterize the extract’s composition. Treatment with *O. aurita* extract significantly increased melanin content in B16-F0 cells by 45% (*p* < 0.01) compared to control. Tyrosinase activity was elevated by 38% after 24 h (*p* < 0.01), with gene and protein expression analyses confirming upregulation of Tyrosinase (TYR) after 0.5 h, Tyrosinase Related Protein-1 (TRP1) after 1 h, and Tyrosinase Related Protein-2 (TRP2) after 8 h. The extract also enhanced the cellular antioxidant environment, as evidenced by increased levels of metabolic cofactors and pigment-precursor amino acids. *O. aurita* methanolic extract accelerates and sustains melanin synthesis and tyrosinase activity, distinguishing its effect from single-compound inducers. These findings support the therapeutic potential of *O. aurita* for pigmentary disorders and skin health. Further studies should investigate its efficacy and safety in vivo and explore its application in cosmeceutical and nutraceutical formulations.

## 1. Introduction

Ultraviolet (UV) radiation exposure is harmful, damaging DNA in skin cells and increasing the risk of skin cancer, such as melanoma [1]. Melanin, a pigment synthesized in melanocytes, does exert a fundamental photoprotective activity in human skin, absorbing and scattering UV radiation [2]. Melanin is a pigment with elevated antioxidant capacity [3] able to neutralize free radicals, protecting skin cells from oxidative stress [2,3].

Melanin biosynthesis modulation [4] can lead to hyperpigmentation (e.g., melasma, age spots) or hypopigmentation (e.g., *vitiligo*) [4]. *Vitiligo* is an autoimmune disease characterized by a loss of pigmentary melanocytes, which results in white macules and patches on the skin, commonly affecting the hands, face, and feet [5].

Vitamins and mineral deficiencies may have a role in *vitiligo*’s development [6]. Low serum levels of copper or zinc and 25-hydroxyvitamin D levels are observed in children with *vitiligo* [7]. Yet, people affected by *vitiligo* present a high oxidative stress index and total serum oxidant status [5], in concomitance with the low levels of functional glutathione peroxidase [8]. Yet, the lack of melanin increases the risks of skin cancer [8].

To counteract *vitiligo* disease [9], the widely used therapies are based on topical application of corticosteroids or calcineurin inhibitors administration and/or on narrowband ultraviolet B monotherapy [9]. These therapies are quite expensive with potential side effects [10]; interest in natural products able to intercept the inhibition of melanogenesis is increasing [11]. Also, research on antioxidant molecules aiming to counteract the oxidative stress in melanocytes is worthy of attention [6]. One of the most effective targets in this frame is the tyrosinase activity, its involvement in melanin accumulation being well known [12]. Higher plants already provide resources for the treatment of melanin deficiency [12]. *Piper nigrum* extract, or the pure alkaloid piperine, has effects in the treatment of *vitiligo* [13]. Piperine stimulates the proliferation of melanocytes and increases the number and length of cell dendrites [8]. While piperine induces a diffuse pigmentation, treatment with *Piper nigrum* extract is more rapid and results in pigmentation islands [8].

Marine photosynthetic microorganisms, representing a crucial resource for bioactives’ production [14], are not explored as melanin inducers, while some microalgae have been reported to have whitening activity [15]. In this context, diatoms are an appealing resource for biotechnology [16,17], even though they have been little exploited so far. Diatoms contain bioactive xanthophylls, such as fucoxanthin, with known antioxidant, anti-inflammatory, anti-diabetes, and anti-cancer activities [18], and diatoxanthin (Dt), a molecule with antioxidant, anti-inflammatory, and chemopreventive activities [19,20,21].

The objective of this study was to explore the in vitro capacity of a diatom, *Odontella aurita*, to modulate the melanogenesis pathway and to disentangle its action mechanism and intracellular signaling in the *B16-F0* cell line. Our study developed a comprehensive analysis and reports on the molecular mechanisms, such as the regulation of key genes and proteins underlying the induction of melanogenesis, together with a biochemical and metabolic characterization of *O. aurita* biomass. The marine diatom *O. aurita* resource was selected as being the unique diatom authorized as a food supplement in the EU [22]. Also, it is rich in polysaccharides, carotenoids, and lipids [23,24] with known antioxidant and anti-inflammatory properties [23]. The murine *B16-F0* cell line is a well-established model for studies concerning melanogenic principles [25].

This in vitro study indicates that *O. aurita* extract acts as an enhancer of melanin synthesis and opens avenues for further in vivo exploration, which is essential for potential applications in cosmetics targeting *vitiligo* and other skin disorders. Additionally, given its unique status as a diatom authorized as a food supplement in the EU and its rich composition of bioactive compounds with in vitro antioxidant and anti-inflammatory properties, *O. aurita* holds promise for use in a food supplement aimed at the prevention of skin diseases, further broadening its therapeutic and preventive potential.

Given the growing interest in natural and sustainable solutions for skin health, the investigation of marine diatoms as modulators of melanogenesis represents a promising frontier. By leveraging their unique biochemical composition and bioactive compounds, such as xanthophylls and polysaccharides, these microorganisms offer novel opportunities for developing alternative therapies. This study aims to bridge the gap in current research by providing detailed insights into the efficacy and mechanisms of *O. aurita* extract in enhancing melanin synthesis, thereby contributing valuable knowledge to both scientific and therapeutic communities.

## 2. Materials and Methods

### 2.1. Odontella Aurita Cultivation

The diatom *Odontella aurita* (strain K-1252) was purchased from NORCAA (the Norwegian Culture Collection of Algae) and was grown in a 3 L glass aquarium in triplicate at 20 °C. Culture medium was prepared with filtered (through 0.7 µm GF/F glass fiber) and autoclaved seawater amended with enriched F/2 medium [26]. 

The light conditions were established with a custom-built LED illumination system [26], utilizing a photoperiod of 12:12 h dark/light with a sinusoidal light distribution, a midday light intensity peak of 90 µmol photons s−1 m−2, and a spectral composition (R:G:B) of 05:40:55%. These cultivation and experimental setups were performed exclusively in vitro; no in vivo studies were conducted in this phase of the research.

### 2.2. Experimental Strategy

The cultivation described here was carried out under controlled laboratory conditions, and all subsequent experimental procedures—including extraction and testing for effects on melanogenesis—were conducted in vitro, using murine and human cell culture models.

From the biomass harvested, methanolic extraction was carried out, and its ability to intercept the melanogenesis process in the murine melanoma cell line *B16-F0* was explored. Also, a comparative analysis using tangeretin and fucoidan was undertaken. Tangeretin (4′,5,6,7,8-pentamethoxyflavone) is a well-known melanogenesis inducer and was considered as a positive control, while fucoidan, a well-known melanogenesis inhibitor of marine origin (from *Fucus vesiculosus*), represented the negative control.

Production of intracellular melanin in *B16-F0* cells in vitro was monitored throughout a time-course experiment (from 0 to 96 h), while the tyrosinase activity was assessed over time until 48 h, together with the expression of genes and proteins involved in melanogenesis.

In parallel, the biochemical, metabolic, and antioxidant characterization of the *O. aurita* biomass was performed. Biochemical assessment concerned the determination of carotenoids, vitamins (D_2_, D_3,_ and E), total phenolic content (TPC), total flavonoid content (TFC), and zinc concentration. The radical scavenging ability and antioxidant-related capacities of *O. aurita* were estimated through ABTS, FRAP, and ORAC assays. Metabolomics using nuclear magnetic resonance (NMR) spectroscopy was developed to determine the metabolic profile of *O. aurita*.

### 2.3. Methanolic Extract Preparation

The methanolic extract of *O. aurita* was obtained by dissolving 500 mg of dried biomass in 6 mL of methanol and then sonicating for 90 s in pulse mode (30 s ON–30 s OFF–30 s ON) at 20% of amplitude by using a SONOPULS HD 2070.2 (BANDELIN, Berlin, Germany). After sonication, the sample was centrifuged at 3900× *g* for 15 min at 4 °C using a Sigma 3-18KS (Sigma Laborzentrifugen, Osterode am Harz, Germany), and the supernatant was transferred into a glass vial. The liquid methanolic phase was completely evaporated by rotary evaporation at 37 °C using a Rotavapor R-100 (Büchi Labortechnik, Postfach, Switzerland). After evaporation, the dried extract, methanol-free, was weighed and stored at −20 °C.

### 2.4. Murine and Human Cell Lines

The murine *B16-F0* melanoma cell line was purchased from the ATCC (Cat. No. CRL-6322, Manassas, VA, USA) and grown in DMEM/F-12 supplemented with 10% (*v*/*v*) Fetal Bovine Serum (FBS), 100 units mL^−1^ penicillin, 100 units mL^−1^ streptomycin, and 2 mM of L-glutamine. The *B16-F0* cells were cultured in a Forma™ Series II Water-Jacketed CO_2_ Incubator (Cat. No. 3111, Thermo Scientific, Waltham, MA, USA) with a 5% CO_2_ atmosphere at 37 °C.

The human epidermal melanocytes (HEMa) cell line was purchased from the ATCC (Cat. No. PCS-200-013, Manassas, VA, USA) and grown in HEM Growth Medium (Cat. No. 135-500, Cell Applications, San Diego, CA, USA). The HEMa cells were cultured in a Forma™ Series II Water-Jacketed CO_2_ Incubator (Cat. No. 3111, Thermo Scientific, Waltham, MA, USA) with a 5% CO_2_ atmosphere at 37 °C.

### 2.5. Cell Viability Assessment

Cells (2 × 10^3^ cells well^−1^, 100 μL well^−1^) from the two cell lines were seeded in two different 96-well plates (TPP Techno Plastic AG, Trasadingen, Switzerland) and kept overnight for attachment. Cells were then treated with different concentrations of the methanolic extract of *O. aurita* (0.1–1–1.25–1.5–3–10–50–100 µg mL^−1^), while DMSO (1%, used as a vehicle) was used as a control (untreated cells). Moreover, the *B16-F0* cells were also treated by tangeretin (6–13–25–50 µM; CAS No. 481-53-8; Cat. No. T8951, Sigma-Aldrich, St. Louis, MO, USA) or fucoidan (0.1–10–100 µg mL^−1^; CAS No. 9072-19-9; Cat. No. F5631, Sigma-Aldrich, St. Louis, MO, USA). At the end of the 48 h incubation, cell viability was estimated using 3-(4,5-dimethylthiazol-2-yl)-2,5-diphenyltetrazolium bromide (MTT; A2231, AppliChem, Darmstadt, Germany). Cells were incubated for 3 h at 37 °C with MTT (final concentration of 0.5 mg mL^−1^). The resulting formazan crystals, only produced by viable cells, were dissolved in 100 µL of isopropyl alcohol. The absorbance was recorded using a Microplate Reader: BioTek Synergy HTX Multimode Reader (Agilent, Santa Clara, CA, USA) at a wavelength of 570 nm. Results were represented as the mean ± SD of the percent of metabolically active cells and estimated as the ratio between the absorbance of each sample and the absorbance of the control (untreated cells). Each experiment was performed in triplicate.

### 2.6. Gene Expression Analysis

The selected genes were tyrosinase (*TYR*, 606933), tyrosinase-related protein 1 (*TRP1*, 115501), and tyrosinase-related protein 2 (*TRP2*, 191275), while the gene used as a control was actin-beta (*ACTB*, 102630), whose expression was found unchanged in control and treated cells. The gene expression was monitored throughout a time course experiment by sampling cells at different times: 0.25 h, 0.5 h, 1 h, 2 h, 4 h, 8 h, and 24 h.

Gene expression levels were calculated using the comparative threshold cycle (ΔΔCt) method. For each sample, the cycle threshold (Ct) value of the gene of interest was first normalized to the Ct value of the reference gene (*ACTB*) to obtain the ΔCt. The ΔCt of treated samples was then compared to the ΔCt of control samples (untreated cells) to determine the ΔΔCt. Relative gene expression was quantified as 2−ΔΔCt, representing the fold change in expression relative to the control group. All calculations were performed using data obtained from triplicate experiments to ensure reliability and reproducibility.

The *B16-F0* cells (2 × 10^6^ cells well^−1^, 2 mL well^−1^) were seeded in 6-well plates (TPP Techno Plastic AG, Trasadingen, Switzerland) and kept overnight for attachment. Cells were then treated with the *O. aurita* methanolic extract at a concentration of 10 µg mL^−1^, tangeretin (50 µM), or fucoidan (10 µg mL^−1^), while DMSO (1%) was used as a control.

During sampling, the medium was removed, and the attached cells were rapidly washed with cold phosphate-buffered saline (PBS) solution and lysed directly in plates by adding 500 µL of TRIsure™ reagent (Cat. No. BIO-3803, Meridian Bioscience, Cincinnati, OH, USA), used for RNA extraction, following the manufacturer’s protocol. RNA concentration and purity were assessed using the NanoDrop 1000 UV-Vis Spectrophotometer (Thermo Fisher Scientific, Waltham, MA, USA). The reverse transcription reaction was carried out using the iScript cDNA Synthesis Kit (Cat. No. 1708090, Bio-Rad, Hercules, CA, USA) following the manufacturer’s protocol. Real-time quantitative polymerase chain reaction (RT-qPCR) was performed to assess the effect of treatment on the expression of the targeted genes. Primers’ design (Table S1) and validation were performed for the assessment of RT-qPCR experiments.

RT-qPCR experiments were performed in triplicate using a MyTaq™ Red Mix kit (Cat. No. BIO-25043, Meridian Biosciences Inc., Cincinnati, OH, USA). The 384-well plates for RT-qPCR were run on a ViiA 7 Real-Time PCR System (Applied Biosystems, Waltham, MA, USA). The standard fast PCR cycling protocol was run with 10 µL reaction volumes. Cycling conditions were setup in three stages: the first stage corresponded to 50 °C for 2 min followed by 95 °C for 10 min; the second stage consisted in 40 cycles at 95 °C for 15 s and 60 °C for 1 min; the last stage (melt curve) corresponded to 95 °C for 15 s, then 60 °C for 1 min and 95 °C for 15 s. The cycle threshold (Ct)-values were analyzed through PCR array data analysis available online software (https://dataanalysis2.qiagen.com/pcr, (accessed on 30 September 2025) Qiagen, Hilden, Germany).

### 2.7. Protein Expression Analysis

Five key proteins associated with melanogenesis—tyrosinase, tyrosinase-related protein 1 (TRP1), tyrosinase-related protein 2 (TRP2), extracellular signal-regulated kinases 1 (ERK1), and extracellular signal-regulated kinases 2 (ERK2)—were evaluated for their expression profiles at four distinct time points: 0 h, 4 h, 24 h, and 48 h. The B16-F0B16-F0 cells (2 × 106 cells per well, 2 mL per well) were seeded into 6-well plates (TPP Techno Plastic AG, Trasadingen, Switzerland) and incubated overnight to allow for cell attachment. Treatments included O. aurita methanolic extract (10 µg mL^−1^), tangeretin (50 µM), and fucoidan (10 µg mL^−1^), with DMSO (1%) serving as the vehicle control.

At each sampling point, the medium was carefully removed, and cell lysates were prepared by scraping cells into 500 µL of RIPA Lysis and Extraction Buffer (Cat. No. 89900, Thermo Fisher Scientific, Waltham, MA, USA), enhanced with Halt™ Protease and Phosphatase Inhibitor Cocktail (Cat. No. 78442, Thermo Fisher Scientific, Waltham, MA, USA). Lysates were incubated on ice for 15 min and clarified by centrifugation at 14,000× *g* for 20 min. Protein concentrations were quantified using the Bradford assay (Bradford Solution for Protein Determination, Cat. No. A6932, Applichem, Darmstadt, Germany) with bovine serum albumin (BSA, Cat. A2058, Sigma-Aldrich, St. Louis, MO, USA) as the standard. The protein extracts were subsequently stored at −20 °C for later Western blot analysis.

For Western blotting, protein samples were denatured at 95 °C for 5 min, followed by separation via 10% SDS-PAGE. Gels were stained with Coomassie Brilliant Blue R-250 Staining Solution (Cat. No. 161-0436, Bio-Rad, Hercules, CA, USA) or transferred onto Trans-Blot Turbo Midi 0.2 µm Nitrocellulose membranes (Cat. No. 170-4159, Bio-Rad, Hercules, CA, USA) using the Trans-Blot Turbo Transfer System (Cat. No. 170-4150, Bio-Rad, Hercules, CA, USA). Membranes were blocked for 1 h in a solution of Tris Buffered Saline (TBS) containing 0.1% (*v*/*v*) Tween 20^®^ (CAS No. 9005-64-5; Cat No. P1379, Sigma-Aldrich, St. Louis, MO, USA) and 5% (*w*/*v*) BSA.

Following blocking, membranes were incubated overnight at 4 °C with primary antibodies (Antibodies.com, Cambridge, UK) at appropriate dilutions in blocking solution (see Table 1 for details). Anti-β-Actin antibody (Cat. No. 4970, Cell Signaling Technology, Danvers, MA, USA), diluted 1:1000, was used as a positive control. After primary antibody incubation, membranes were washed three times (10 min each) with TBS/Tween solution (0.1%) and incubated for 2 h at room temperature with secondary antibodies: Goat Anti-Mouse HRP conjugated antibody (Cat. No. A17352, Antibodies.com, Cambridge, UK) or Goat Anti-Rabbit HRP conjugated antibody (Cat. No. 31461, Thermo Fisher Scientific, Waltham, MA, USA), both at 1:10,000 dilution in blocking solution. Membranes were again washed three times (5 min each) with TBS/Tween (0.1%).

Detection was carried out using the Clarity Western ECL Substrate (Cat. No. 1705061, Bio-Rad, Hercules, CA, USA), and images were captured with the ChemiDoc™ MP Imaging System (Cat. No. 120-3154, Bio-Rad, Hercules, CA, USA). Densitometric analysis of the immunoreactive bands corresponding to each target protein was performed using ImageLab software Version 6.1.0 build 7(Bio-Rad, Hercules, CA, USA).

All experiments were performed in triplicate to ensure data reliability and reproducibility. The detailed protocols and data acquisition methods described here enable others to replicate the study and validate the findings, thereby supporting transparency in scientific reporting.

### 2.8. Tyrosinase Activity

The enzymatic assay was monitored at 0 h, 4 h, 24 h, and 48 h, using the Tyrosinase Activity Assay Kit, purchased from Abcam (Cat. No. ab252899, Abcam, Cambridge, United Kingdom). The B16-F0 cells (2 × 106 cells well-1, 2 mL well^−1^) were seeded in 6-well plates (TPP Techno Plastic AG, Trasadingen, Switzerland), kept overnight for attachment, and then incubated with O. aurita methanolic extract (10 µg mL^−1^), tangeretin (50 µM) or fucoidan (10 µg mL^−1^), while DMSO (1%) was used as a control (untreated condition). During sampling, the medium was removed, and the attached cells were rapidly washed with cold phosphate-buffered saline (PBS) solution. After removing PBS, cells were lysed directly in the well by using 450 µL of cold Tyrosinase Assay Buffer (supplied in the kit) and incubated for 10 min on ice. Then, lysates were clarified by centrifuging at 10,000× *g* for 15 min at 4 °C, and the supernatants were transferred into fresh tubes and stored at −80 °C. The total protein concentration was determined by performing a Bradford Assay using Bradford Solution for Protein Determination (Cat. No. A6932, Applichem, Darmstadt, Germany), with bovine serum albumin (BSA, cat. A2058, Sigma-Aldrich, St. Louis, MO, USA) used as a standard. The enzymatic assay was performed according to the manufacturer’s protocol, using samples at a protein concentration of 10 µg µL^−1^. The assay was performed in triplicate, and the absorbance of the samples was read using a Microplate Reader: BioTek Synergy HTX Multimode Reader (Agilent, Santa Clara, CA, USA) at a wavelength of 510 nm. The tyrosinase-specific activity was expressed as units of enzyme per mg of protein (U mg protein-1).

Additionally, enzymatic tests with purified tyrosinase were conducted to determine whether the *O. aurita* methanolic extract interferes with the reagent or the enzymatic activity of tyrosinase. The results demonstrated that the extract did not inhibit tyrosinase function nor alter the reagent’s response, thereby confirming the specificity of the assay and ruling out any non-specific interference.

### 2.9. Melanin Content

To determine the extracellular melanin content, the culture medium was collected after 48 h and centrifuged at 10,000× *g* for 10 min at 4 °C to remove any cell debris. The supernatant was carefully transferred to clean tubes. The melanin content in the supernatant was quantified by measuring the absorbance at 405 nm using a microplate reader (BioTek Synergy HTX Multimode Reader, Agilent, Santa Clara, CA, USA). A standard curve was prepared using known concentrations of pure melanin (Sigma-Aldrich, St. Louis, MO, USA) dissolved in 0.1 M NaOH, allowing the extracellular melanin concentration in each sample to be calculated based on its absorbance. All measurements were performed in triplicate, and results were expressed as micrograms of melanin per milliliter of culture medium (μg mL^−1^).

Intracellular melanin content was quantified at 0 h, 4 h, 24 h, 48 h, 72 h, and 96 h post-incubation with *O. aurita* methanolic extract (10 µg mL^−1^), tangeretin (50 µM), or fucoidan (10 µg mL^−1^); DMSO (1%) served as the control (untreated condition). Experiments were performed in triplicate. B16-F0 cells (2 × 103 cells well^−1^, 100 μL well^−1^) were seeded in 96-well plates (TPP Techno Plastic AG, Trasadingen, Switzerland) and allowed to attach overnight. Cells were rapidly washed in phosphate-buffered saline (PBS) and detached by trypsinization. Cell suspensions were transferred into fresh tubes and centrifuged at 500 rpm for 5 min. Supernatants were discarded, and cell pellets were resuspended in 1 N NaOH containing 0.2 mM phenylmethanesulfonyl fluoride (PMSF, CAS No. 329-98-6; Cat. No. 93482, Sigma-Aldrich, St. Louis, MO, USA) and 0.1% (*v*/*v*) Tween 20^®^ (CAS No. 9005-64-5; Cat No. P1379, Sigma-Aldrich, St. Louis, MO, USA). Suspensions were incubated for 1 h at 80 °C, then centrifuged at 15,000 rpm for 15 min at 4 °C. Aliquots (100 µL) of the supernatant were transferred to transparent flat-bottom 96-well plates (TPP Techno Plastic AG, Trasadingen, Switzerland), and absorbance was measured at 490 nm using a BioTek Synergy HTX Multimode Reader (Agilent, Santa Clara, CA, USA). Synthetic melanin (CAS No. 8049-97-6; Cat. No. M8631, Sigma-Aldrich, St. Louis, MO, USA) was used as the standard for melanin concentration estimation (µg mL^−1^).

To assess potential background signal arising from the *O. aurita* methanolic extract, control experiments were conducted in which the extract was incubated under assay conditions in the absence of cells. Absorbance readings at 490 nm (melanin assay) and 510 nm (tyrosinase activity assay) confirmed negligible background signal, indicating the extract did not contribute to absorbance at the relevant wavelengths.

Assay recovery and linearity were evaluated by spiking known quantities of synthetic melanin into the *O. aurita* extract matrix and performing the quantification protocol as described. Measured melanin concentrations demonstrated linearity across the tested range, with recovery rates consistently exceeding 95%. These results confirmed that the extract matrix does not interfere with the spectrophotometric quantification of melanin, and standard curves generated in the presence of extract were indistinguishable from those in buffer alone.

### 2.10. Carotenoids

Carotenoid analysis was carried out with HPLC. A 10 mg aliquot of dried *O. aurita* biomass was mechanically ground in 3 mL of absolute methanol for 3 min, before being sonicated for 90 s in pulse mode (30 s ON–30 s OFF–30 s ON; SONOPULS HD 2070.2 (BANDELIN, Berlin, Germany)). The resulting homogenate was filtered through Whatman 25 mm GF/F filters, and the volume of the filtrate was carefully recorded. A 500 µL portion of the pigment extract was combined with 250 µL of 1 M ammonium acetate and incubated for 5 min in the dark at 4 °C. The extract was injected into a 50 µL loop mounted on a Hewlett Packard series 1100 HPLC system. The reversed-phase column was a 2.6 mm diameter C8 Kinetex column (50 × 4.6 mm; Phenomenex, Torrance, CA, USA), and the mobile phase consisted of a solution A (methanol:0.5 N aqueous ammonium acetate, 70:30 *v*/*v*) and B (absolute methanol). The flow rate was set at 1.7 mL min^−1^, and during the 12 min elution, the solvent gradient was programmed as follows: 75% A at 0 min, 50% A at 1 min, 0% A at 8 min, 0% A at 11 min, and 75% A at 12 min. Pigments were detected at 440 nm using a Hewlett Packard photodiode array detector (model DAD series 1100), which provided spectra from 400 to 700 nm for each pigment detected. Identification of pigments was based on their retention time and absorption spectra, while quantification was achieved using pure standards sourced from D.H.I. Water and Environment (Hørsholm, Denmark).

### 2.11. Total Phenolic Content and Total Flavonoid Content

The estimation of the total phenolic content (TPC) and total flavonoid content (TFC) of *O. aurita* biomass was performed by slightly modifying the protocols previously described (26). An aliquot of 3 mg of dried microalgal biomass was dissolved in 500 μL of methanol and then sonicated for 90 s in pulse mode (30 s ON–30 s OFF–30 s ON) at 20% of amplitude by using a SONOPULS HD 2070.2 (BANDELIN, Berlin, Germany). The samples were centrifuged at 3900× *g* for 15 min at 4 °C using a Sigma 3-18KS (Sigma Laborzentrifugen, Osterode am Harz, Germany), and the supernatants were transferred into fresh tubes.

For TPC estimation, an aliquot of 150 μL of the supernatant was added into each well of a transparent 96-well plate (TPP Techno Plastic Products AG, Trasadingen, Switzerland) and mixed with 100 μL of Folin–Ciocalteu’s Reagent (Cat. No. A5084, AppliChem GmbH, Darmstadt, Germany) previously diluted 1:10 (*v*/*v*) in H_2_O. After 4 min of incubation at room temperature (RT), 80 μL of sodium carbonate (Na_2_CO_3_, CAS No. 497-19-8; Cat. No. 131648, AppliChem GmbH, Darmstadt, Germany) solution (75 g L^−1^ concentrated in H_2_O) was added to each well. After 1 h of incubation at RT in the dark, the absorbance was read at 765 nm using a BioTek Synergy HTX Multimode Reader (Agilent, Santa Clara, CA, USA) to estimate the content of phenolic compounds, using gallic acid ((HO)_3_C_6_H_2_CO_2_H, CAS No. 149-91-7; Cat. No. G7384, Merck, Darmstadt, Germany) as the standard. The standard phenolic compound typically used in the determination of total phenolic content (TPC) is gallic acid. The assay was performed in triplicate, and the concentrations were expressed as gallic acid equivalents (GAE) per mg DW^−1^.

Results are usually expressed following common protocols in phenolic content estimation.

For TFC estimation, an aliquot of 100 μL of the 1/20 (*v*/*v*) pre-diluted sample (80% methanol *v*/*v* in H_2_O) was mixed with 100 μL of 2% solution (*w*/*v* in H_2_O) of aluminum chloride (AlCl_3_, CAS No. 7446-70-0; Cat. No. 8.01081, Merck, Darmstadt, Germany) into each well of a transparent 96-well plate (TPP Techno Plastic Products AG, Trasadingen, Switzerland).

The standard flavonoid compound typically used for the determination of total flavonoid content (TFC) is quercetin.

After 30 min of incubation in the dark at RT, the absorbance was read at 410 nm using a BioTek Synergy HTX Multimode Reader (Agilent, Santa Clara, CA, USA) to estimate the content of flavonoid compounds, using quercetin (C_15_H_10_O_7_, CAS No. 117-39-5; Cat. No. Q4951, Merck, Darmstadt, Germany) as the standard. The assay was performed in triplicate, and the results were expressed as quercetin equivalents (QE) per mg DW^−1^.

### 2.12. Vitamins Determination

An aliquot of 5 mg of dried biomass was dissolved in 500 μL of RIPA Lysis and Extraction Buffer (Cat. No. 89900, Thermo Fisher Scientific, Waltham, MA, USA) and sonicated for 90 s in pulse mode (30 s ON–30 s OFF–30 s ON) at 20% of amplitude by using a SONOPULS HD 2070.2 (BANDELIN, Berlin, Germany). Samples were then centrifuged at 13,000× g for 5 min at 4 °C using a Sigma 3-18KS (Sigma Laborzentrifugen, Osterode am Harz, Germany), and the supernatants were transferred into new tubes. The content of vitamins C, D_2_, D_3_ and E was determined by performing a competitive ELISA assay following the protocol reported in [26], using specific antibodies (Table 2).

The vitamin content was quantified by referring to the calibration curve obtained by using pure vitamins as the standard. The assay was performed in triplicate, and the results were expressed as mean ± SD of ng of vitamin on mg of dry weight (ng mg DW^−1^).

### 2.13. Zinc Determination

An aliquot of 3 mg of dried biomass was dissolved in 500 μL of RIPA Buffer Lysis and Extraction Buffer (Cat. No. 89900, Thermo Fisher Scientific, Waltham, MA, USA) and sonicated for 90 s in pulse mode (30 s ON–30 s OFF–30 s ON) at 20% of amplitude by using a SONOPULS HD 2070.2 (BANDELIN, Berlin, Germany). The samples were centrifuged at 13,000× *g* for 5 min at 4 °C using a Sigma 3-18KS (Sigma Laborzentrifugen, Osterode am Harz, Germany), and the supernatants were transferred into new tubes. The cell lysates were deproteinized by adding an equal volume of 7% trichloroacetic acid (TCA) solution and centrifuging at 13,000× *g* for 5 min at 4 °C. Samples were neutralized by adding 1 M sodium carbonate (Na_2_CO_3_, CAS No. 497-19-8; Cat. No. 131648, AppliChem GmbH, Darmstadt, Germany) solution, then vortexing and incubating on ice for 5 min. Zinc concentration was estimated by using the Zinc Quantification Kit (Cat. No. ab176725, Abcam, Cambridge, United Kingdom), according to the manufacturer’s protocol. The assay was performed in a black 96-well plate (Cat. No. 3694, Corning Inc., Corning, NY, USA), and the fluorescence was measured using Infinite^®^ M1000 PRO (Ex: 485 nm, Em: 525 nm; TECAN, Männedorf, Switzerland). The zinc concentration was estimated by referring to the calibration curve obtained by using zinc chloride (ZnCl_2_) supplied with the kit. The assay was performed in triplicate, and the results were expressed as mean ± SD of pmol of Zn^2+^ per mg of dry weight (pmol mg DW^−1^).

### 2.14. Antioxidant Capacity (ABTS, FRAP, ORAC)

The antioxidant capacity was performed on the methanolic extract.

The ABTS (2,2′-azinobis(3-ethylbenzothiazoline-6-sulphonic acid)) assay, allowing for testing of the ability of the extract to scavenge ABTS^+^ radical, was performed following the protocol described in [26]. The results were expressed as mean ± SD of µg of Trolox equivalents (TE) per mg of dry weight (dried biomass; mg DW^−1^). The FRAP (Ferric Reducing Antioxidant Power) assay, assessing the reductive antioxidant properties of the extract, was performed following the protocol described in [26]). The results were expressed as mean ± SD of µg of Trolox equivalents (TE) on mg of dry weight (dried biomass; mg DW^−1^). The ORAC (Oxygen Radical Absorbance Capacity) assay, assessing the chain-breaking capacity of the extract, was performed following the protocol described in [26]. The results were expressed as mean ± SD of µg of Trolox equivalents (TE) per mg of dry weight (dried biomass; mg DW^−1^).

### 2.15. NMR-Based Metabolic Profile

The molecular content of the *O. aurita* biomass was acquired through Nuclear Magnetic Resonance (NMR) spectroscopy, and the full spectrum was assigned. Combined extraction of polar and lipophilic metabolites was carried out using methanol/water/chloroform as suggested [27]. Polar and nonpolar fractions were transferred into different glass vials, and the solvents were removed by using a rotary vacuum evaporator at room temperature. Samples were then stored at −80° C until the spectroscopic analysis. Hydrophilic fractions were resuspended in 630 µL of phosphate-buffered saline (PBS, pH 7.4), adding 70 µL of ^2^H_2_O solution (containing 1 mM sodium 3-trimethylsylyl (2,2,3,3-2H4) propionate (TSP) as a chemical shift reference for ^1^H spectra) to provide a field frequency lock, reaching 700 µL of total volume. Samples were then loaded into the autosampler at 4 °C, and NMR spectra were acquired on a Bruker Avance III–600 MHz spectrometer (BrukerBioSpin GmbH, Rheinstetten, Germany), equipped with a TCI CryoProbe fitted with a gradient along the *Z*-axis, at a probe temperature of 27 °C. In particular, standard 1D proton spectra and 2D experiments (clean total-correlation spectroscopy TOCSY with DIPSI pulse scheme and heteronuclear single quantum coherence HSQC) were acquired, providing monodimensional metabolic profiles and homonuclear and heteronuclear spectra for metabolite identification. Metabolite assignments were achieved by comparing signal chemical shifts with the literature (Fan WMT1996) [28] and online databases [29].

### 2.16. Statistical Analysis

All experiments were conducted with a minimum of three independent biological replicates to ensure reproducibility and reliability of the results. Data are presented as mean values ± standard deviation (SD) unless otherwise specified. Statistical significance was assessed using one-way analysis of variance (ANOVA) to identify differences among groups. Where applicable, post hoc multiple comparison tests, such as Dunnett’s or Tukey’s test, were applied to determine specific group differences following ANOVA. A *p*-value of less than 0.05 was considered statistically significant throughout the analysis.

All statistical analyses were performed using GraphPad Prism (version 10, GraphPad Software, San Diego, CA, USA).

## 3. Results

### 3.1. B16-F0 and HEMa Cells Viability

**Figure 1:**
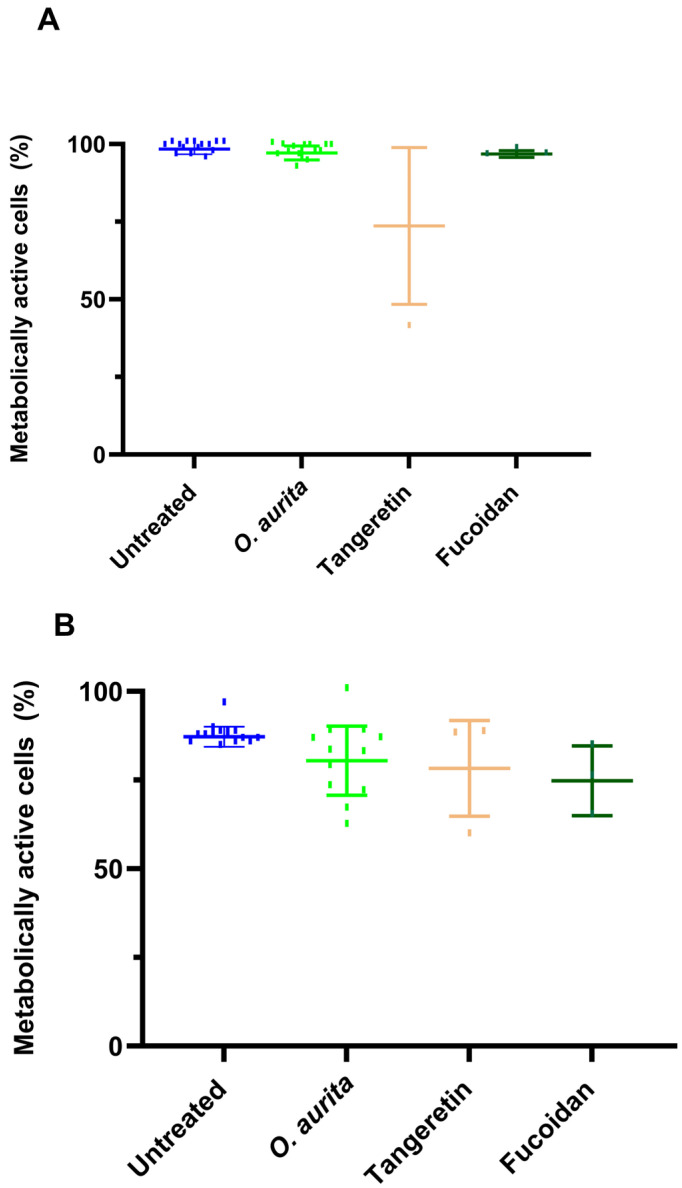
Assessment of cell viability in *B16-F0* murine melanoma cells and HEMa human epidermal melanocytes following treatment with *O. aurita* methanolic extract, fucoidan, and tangeretin. (**A**) *B16-F0* cells were exposed to increasing concentrations of *O. aurita* extract (0.1–0.5–0.75–1–1.25–1.5–3-5–7.5–10-25–50-75 and 100 µg mL^−1^); fucoidan: 0.1–1 and 10 µg mL^−1^, and tangeretin: 6, 13, 25, and 50 µM. Cell viability was quantified using the MTT assay and is expressed as a percentage relative to untreated control cells. (**B**) HEMa cell viability was measured under the same treatment conditions to evaluate cytotoxicity in non-murine cells. Data represent SD from at least three independent experiments. Statistical significance was determined by one-way ANOVA followed by Dunnett’s or Tukey’s post hoc test where appropriate.

The dried *O. aurita* methanolic extract did not induce any cytotoxic effect on the murine *B16-F0* cells (*p* > 0.05; Figure 1A) at concentrations up to 100 µg mL^−1^. Fucoidan, tested at 10 µg mL^−1^, did not affect B16-F0 cell viability (*p* > 0.05; Figure 1A) either, while the melanogenesis inducer tangeretin, assessed at concentrations up to 50 µM, significantly affected *B16-F0* cell viability in a dose-dependent manner (Figure 1A).

The concentrations of tangeretin (up to 50 µM) and fucoidan (10 µg mL^−1^) were selected based on the previous literature [30], indicating their efficacy in modulating melanogenesis without inducing cytotoxicity in similar cell models. These concentrations were chosen to ensure a measurable biological effect while maintaining cell viability, as confirmed by the absence of cytotoxicity observed in the MTT assays reported in this study (Figure 1A). The selected doses also align with established protocols used in related melanogenesis and cell viability studies, providing a reliable basis for comparison and interpretation of results within the experimental framework.

Tangeretin and fucoidan were used as positive and negative controls of melanogenesis induction, respectively.

HEMa (human epidermal melanocyte) cells were included as a negative control to evaluate the cytotoxicity of the *O. aurita* methanolic extract, ensuring that any observed effects on cell viability were not specific to murine cells and confirming the extract’s general safety profile. However, HEMa cells could not be employed to investigate the mechanistic effects of the extract on melanogenesis, as these cells did not exhibit upregulation of melanogenesis-related pathways in response to the treatments [31]. This limitation means that, while HEMa cells are suitable for cytotoxicity assessment, they do not provide a relevant model for studying the modulation of melanogenic mechanisms by the extract, which necessitates the use of B16-F0 murine melanoma cells, where such pathways are responsive and can be experimentally manipulated.

The *O. aurita* methanolic extract was evaluated for its cytotoxic effects on HEMa human epidermal melanocytes across a range of concentrations (1, 10, 25, 50, and 100 µg mL^−1^). At the lower concentrations (1, 10, and 25 µg mL^−1^), there was no significant impact on cell viability, with values remaining comparable to the tangeretin treatment group. However, at the two highest concentrations tested (50 and 100 µg mL^−1^), a slight but statistically significant reduction in cell viability was observed (*p* ≤ 0.01; Figure 1B). This indicates that while *O. aurita* extract is generally non-cytotoxic at lower doses, higher concentrations may exert mild cytotoxic effects on HEMa cells.

### 3.2. Modulation of the Expression of Genes Involved in Melanogenesis in B16-F0 Cells

**Figure 2:**
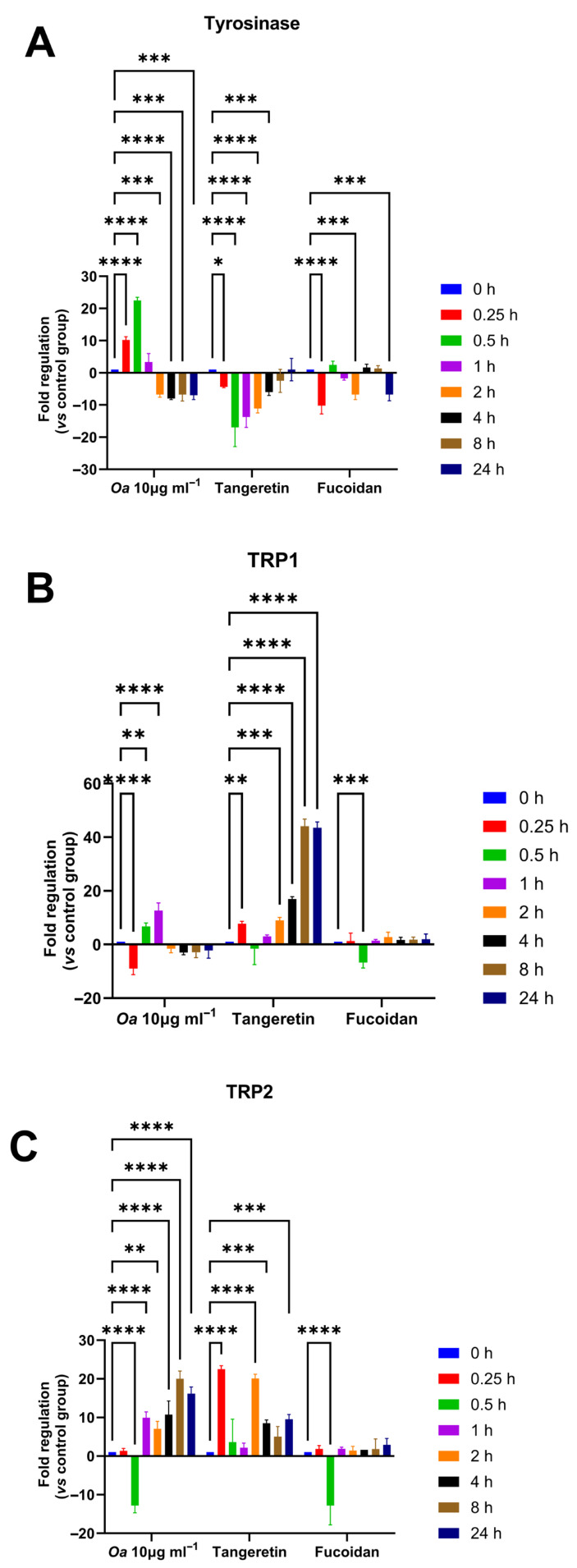
Time-course analysis of the expression levels of genes involved in melanogenesis—specifically tyrosinase (*TYR*, panel A), tyrosinase-related protein 1 (*TRP1*, panel B), and tyrosinase-related protein 2 (*TRP2*, panel C)—in B16-F0 cells following treatment with three different agents: (**A**) *O. aurita* methanolic extract (10 µg mL^−1^), (**B**) tangeretin (50 µM), and (**C**) fucoidan (10 µg mL^−1^). Gene expression was assessed at multiple times: 0, 0.25, 0.5, 1, 2-, 4-, 8-, and 24 h post-treatment. The results are presented as mean ± SD (*n* = 3), showing the fold change in gene expression relative to untreated control cells. The fold regulation values were normalized to the expression of the housekeeping gene beta actin (ACTB), ensuring accurate comparison across samples. Statistically significant differences compared to the control were determined using Dunnett’s test, with significance indicated by * (*p* < 0.05), ** (*p* < 0.01), *** (*p* < 0.001) and **** (*p* < 0.0001). Abbreviations: *TYR* = Tyrosinase, *TRP1* = Tyrosinase Related Protein 1, *TRP2* = Tyrosinase Related Protein 2.

The expression of the three genes, tyrosinase (*TYR*), tyrosinase-related protein 1 (*TRP1*), and tyrosinase-related protein 2 (*TRP2*), was modulated by *O. aurita* methanolic extract, with an upregulation shifted in time. Figure 2 illustrates the time-dependent modulation of three key genes involved in melanogenesis—Tyrosinase (*TYR*), Tyrosinase Related Protein 1 (*TRP1*), and Tyrosinase Related Protein 2 (*TRP2*)—in B16-F0 cells following treatment with the *O. aurita* methanolic extract, tangeretin, and fucoidan. In Figure 2A, exposure to the *O. aurita* methanolic extract (10 µg mL^−1^) led to a rapid upregulation of *TYR* gene expression, detectable as early as 0.25 h, peaking at 0.5 h (*p* < 0.0001), before declining from 2 h onwards. *TRP1* (Figure 2B) expression initially decreased at 0.25 h (*p* < 0.0001), followed by a swift upregulation after 0.5 h (*p* < 0.001), reaching its highest level at 1 h (*p* < 0.0001), before returning to a downregulated state after 2 h (*p* < 0.05). *TRP2* gene expression (Figure 2C) was distinctly upregulated after 1 h and remained elevated through 24 h (*p* < 0.0001).

Tangeretin (50 µM) did not induce *TYR* upregulation, but instead predominantly upregulated *TRP1* gene expression at 4 h (at least *p* < 0.05). In contrast, fucoidan (10 µg mL^−1^) did not promote upregulation of the three melanogenesis-related genes at any time point, and in fact caused significant downregulation at certain intervals (*p* < 0.0001).

### 3.3. Modulation of Protein Expression Involved in Melanogenesis in B16-F0 Cells

**Figure 3:**
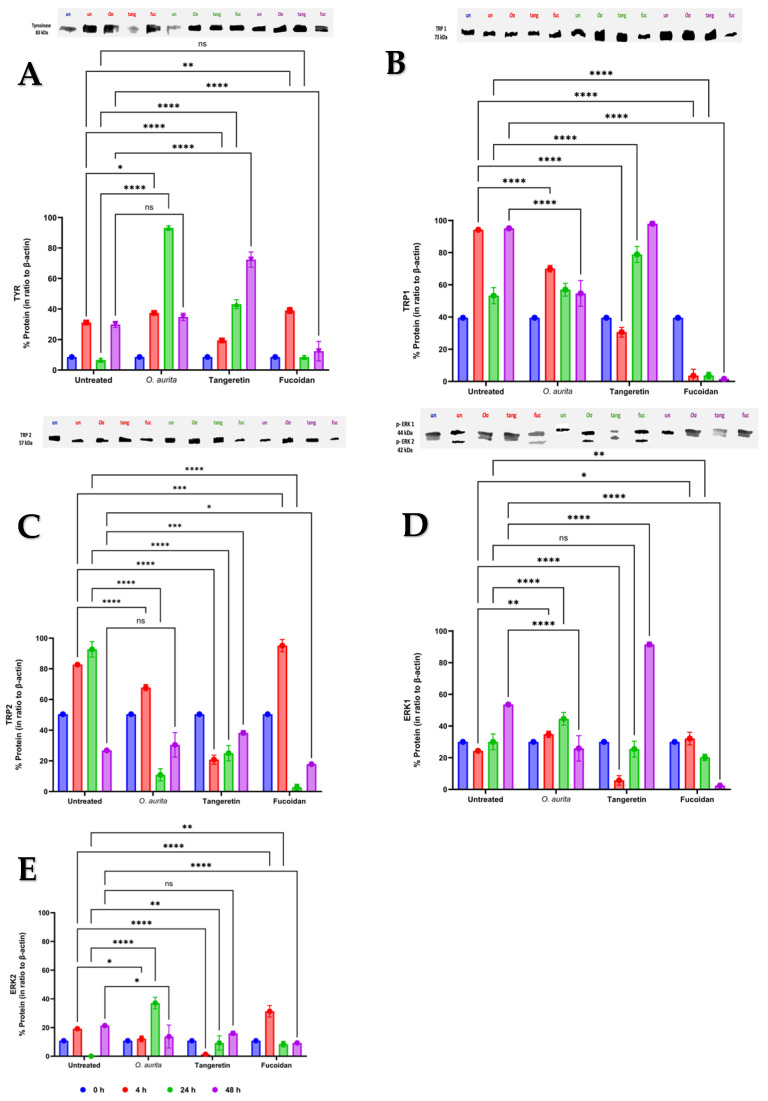
Expression analysis of five melanogenesis-involved proteins in B16-F0 cells (n = 3 biological replicates) treated with *O. aurita* methanolic extract (10 µg mL^−1^), tangeretin (50 µM), or fucoidan (10 µg mL^−1^). The graphs shown at the top of each panel represent a representative example of the electrophoretic blots performed for each protein; the complete, uncropped versions of these blots are available in the Supplementary File. Protein expression levels for (**A**) TYR, (**B**) TRP1, (**C**) TRP2, (**D**) ERK1, and (**E**) ERK2 were monitored at 0 h, 4 h, 24 h, and 48 h. Values are expressed as a percentage of protein normalized to *β*-actin expression levels (mean ± SD, n = 3). Statistical significance—compared to untreated cells—was calculated through Tukey’s test and is indicated by ns (not significant), **** (*p* < 0.0001), *** (*p* < 0.001), ** (*p* < 0.01), and * (*p* < 0.05). TYR = Tyrosinase, TRP1 = Tyrosinase Related Protein 1, TRP2 = Tyrosinase Related Protein 2, ERK1 = Extracellular signal-Regulated Kinase 1, ERK2 = Extracellular signal-Regulated Kinase 2.

Five proteins, tyrosinase (TYR), tyrosinase-related protein 1 (TRP1) and tyrosinase-related protein 2 (TRP2), extracellular signal-regulated kinases 1 (ERK1) and extracellular signal-regulated kinases 2 (ERK2), were targeted (Figure 3A).

Tyrosinase synthesis was overexpressed in the *B16-F0* cells treated with *O. aurita* methanolic extract (10 µg mL^−1^) after 24 h (*p* < 0.0001; Figure 3A) compared to time 0 h and to the untreated cells. Tangeretin increased tyrosinase expression after 48 h (*p* < 0.0001; Figure 3A). Fucoidan decreased tyrosinase synthesis only at time 48 h (*p* < 0.0001, Figure 3A).

The *O. aurita* methanolic extract lowered the expression level of TRP1 compared to untreated cells after 4 and 48 h (*p* < 0.0001; Figure 3B). Tangeretin induced a significant decrease in the expression of TRP1 after 4 h (*p* < 0.0001; Figure 3B) and an overexpression after 24 h (*p* < 0.0001; Figure 3B, reaching expression levels comparable to the untreated cells after 48 h. Fucoidan lowered the expression of TRP1 after 4, 24, and 48 h in comparison with untreated cells (*p* < 0.0001; Figure 3B). TRP2 synthesis was downregulated with *O. aurita* extract or tangeretin (*p* < 0.0001; Figure 3C), except at 48 h, when TRP2 synthesis was comparable to untreated cells (*p* ≥ 0.05; Figure 3C). Fucoidan upregulated the TRP2 expression at 4 h, compared to untreated cells and time 0 h (*p* < 0.001; Figure 3C). Conversely, after 24 h (and still at 48 h) in the presence of fucoidan, the TRP2 expression dropped (*p* < 0.0001; Figure 3C). ERK 1 synthesis was increased by *O. aurita* extract in comparison with time 0 h, and the untreated cells after 4 and 24 h (*p* < 0.001 and *p* < 0.0001, respectively; Figure 3D). ERK 1 synthesis was lowered at time 48 h (*p* < 0.0001; Figure 3D). Tangeretin downregulated the expression of ERK1 at 4 h, while increasing its expression at 48 h, compared to the untreated cells and to time 0 h (*p* < 0.0001; Figure 3D). With fucoidan, ERK1 expression level was downregulated after 24 h and 48 h (*p* < 0.001 and *p* < 0.0001, respectively; Figure 3D).

ERK2 synthesis increased at 24 h with *O. aurita* extract compared to untreated cells and to time 0 h (*p* < 0.0001; Figure 3E). Tangeretin induced a decrease in ERK2 expression at 4 h compared to untreated cells and to time 0 h (*p* < 0.0001; Figure 3E), while fucoidan up-regulated ERK2 synthesis at the same time (*p* < 0.001; Figure 3E). After 24 h, ERK2 synthesis was increased (*p* < 0.001; Figure 3E), while it was downregulated after 48 h (*p* < 0.0001; Figure 3E).

### 3.4. Modulation of Tyrosinase Activity in B16-F0 Cells

**Figure 4:**
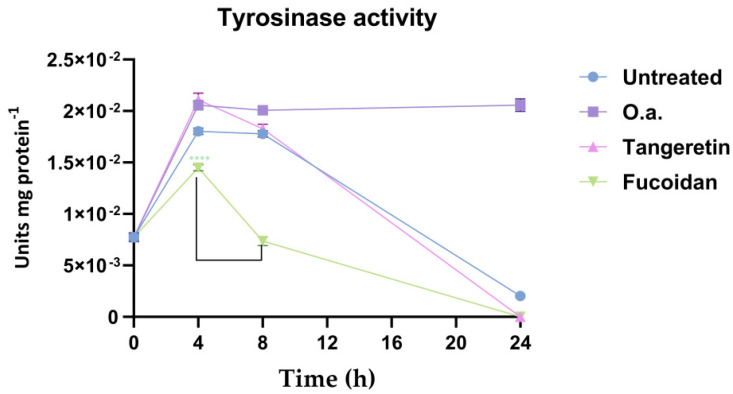
Tyrosinase enzymatic activity in *B16-F0* cells, treated with *O. aurita* methanolic extract (10 µg mL^−1^), tangeretin (50 µM), or fucoidan (10 µg mL^−1^). Tyrosinase activity was assessed at 0 h, 4 h, 8 h, and 24 h. Values are expressed as mean ± SD of tyrosinase enzymatic units per mg of total protein (Units mg protein^−1^). Statistical significance—compared to untreated cells—was calculated through Student’s *t*-test and is indicated by **** (*p* < 0.0001).

The tyrosinase activity initially increased until 4 h in both treated and untreated *B16-F0* cells (Figure 4). In the untreated cells, tyrosinase activity level was almost constant at 4 and 8 h, before dropping to a minimum at 24 h (Figure 4).

In *B16-F0* cells treated with fucoidan, tyrosinase activity was the lowest at each time and dropped down already after 4 h (at least *p* < 0.01; Figure 4).

When treated with tangeretin, the tyrosinase activity in *B16-F0* cells was not stable and decreased after 4 h (Figure 4). Conversely, the tyrosinase activity remained high and stable with time (until 24 h) when cells were treated with *O. aurita* extract (*p* < 0.01; Figure 4).

### 3.5. Extracellular/Intracellular Melanin Synthesis

**Figure 5:**
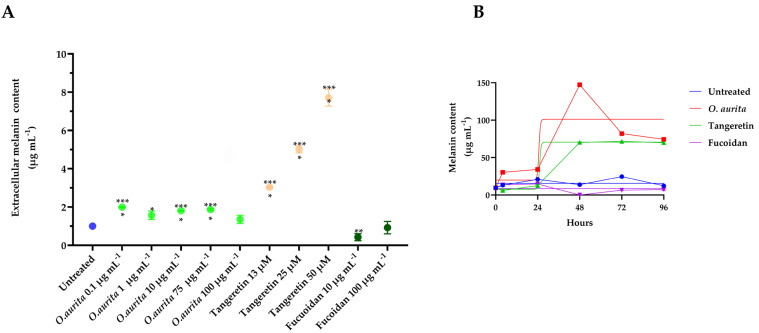
(**A**) Extracellular melanin secretion levels observed in *B16-F0* cells when treated with varying concentrations of *Odontella aurita* extract (1–100 µg mL^−1^), fucoidan (10 and 100 µg mL^−1^), or tangeretin (13–25–50 µM) for 48 h. The graph demonstrates a dose-dependent logarithmic increase in melanin secretion with *O. aurita* extract (0.1–1–10 µg mL^−1^), reaching higher levels than those induced by tangeretin and markedly exceeding the suppression seen with fucoidan. Statistical comparisons to untreated control cells are indicated on the figure by asterisks, representing levels of significance determined by Student’s *t*-test: *** for *p* < 0.0001 and * for *p* < 0.05. The figure visually demonstrates how each treatment affects melanin accumulation over time, highlighting notable increases or decreases relative to controls. (**B**) time-response curve of intracellular melanin synthesis in *B16-F0* cells following treatment with *O. aurita* methanolic extract (10 µg mL^−1^), tangeretin (50 µM), or fucoidan (10 µg mL^−1^). The chart illustrates melanin concentrations measured at six different time points: 0, 4, 24, 48, 72, and 96 h. Data are displayed as the mean ± standard deviation (µg mL^−1^) for each treatment group.

Extracellular melanin secretion in *B16-F0* cells was assessed following treatment with varying concentrations of *Odontella aurita* extract, fucoidan, or tangeretin for 48 h, as illustrated in Figure 5A. The results demonstrated a dose-dependent logarithmic increase in melanin secretion with *O. aurita* extract, particularly at concentrations of 0.1, 1, and 10 µg mL^−1^, which surpassed the levels induced by tangeretin and were markedly higher than the suppression observed with fucoidan. This visual representation highlights that *O. aurita* extract promotes extracellular melanin accumulation more effectively than tangeretin, while fucoidan acts as a suppressor. *O. aurita* extract stimulated the synthesis of melanin in *B16-F0* cells, with the highest intracellular melanin concentration at all time points during the 4-day-long experiment (at least *p* < 0.05; Figure 5B). The peak of melanin was reached at 48 h (150 vs. 14 µg mL^−1^, *p* < 0.001; Figure 5B), before dropping to around 80 µg mL^−1^ (Figure 5B). The treatment with tangeretin increased melanin concentration compared to untreated cells only from 48 h (70 vs. 13 µg mL^−1^, *p* < 0.001; Figure 5B), remaining stable until 96 h (Figure 5B). As expected, treatment with fucoidan did not increase melanin synthesis, lowering it compared to untreated cells from 4 h to 72 h (*p* < 0.05; Figure 5B).

### 3.6. Biochemical Characterization and Antioxidant/Radical Scavenging Activity of O. aurita

The carotenoid profile revealed the high content of the two xanthophylls fucoxanthin (11.16 µg mg DW^−1^) and diadinoxanthin (2.14 µg mg DW^−1^), which contributed to most of the total carotenoid content (TCC; 14.63 µg mg DW^−1^, Table 3). Other carotenoids, such as β-carotene, cis-fucoxanthin, zeaxanthin or violaxanthin were also revealed (Table 3).

The total phenolic content (TPC) and total flavonoid content (TFC) amounted to 18.11 and 1.56 µg mg DW^−1^, respectively (Table 3), while zinc concentration reached 26.09 nmol mg DW^−1^ (Table 3).

The vitamins D_2_ and D_3_ amounted to 385.47 and 15.33 ng mg DW^−1^, respectively (Table 3), with a lower vitamin E content (0.78 ng mg DW^−1^, Table 3). The *O. aurita* methanolic extract exhibited a high specific scavenging activity against ROS, as revealed by the ABTS radical scavenging assay (7.45 µg TE mg DW^−1^, Table 3), compared to absorbance (ORAC assay, 0.33 µg TE mg DW^−1^, Table 3) or reduction capacities (FRAP, 0.13 µg TE mg DW^−1^, Table 3).

### 3.7. Metabolic Profile of O. aurita Methanolic Extract

**Figure 6:**
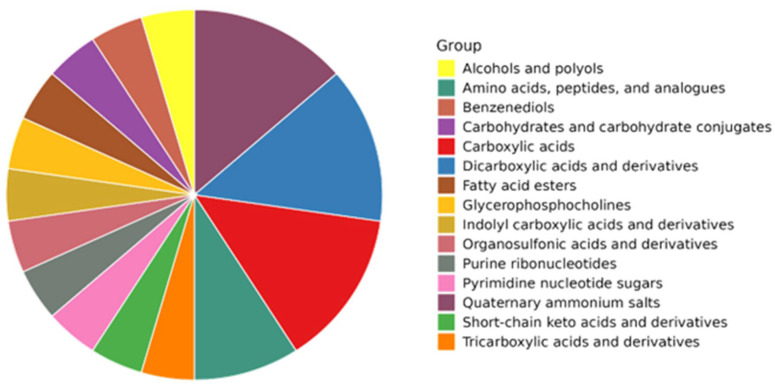
Relative contribution of the main hydrophilic classes of the *O. aurita* methanolic extract revealed by NMR.

The four main classes in terms of relative abundance (50% of the total) were assigned as carboxylic and dicarboxylic acids, carbohydrates or carbohydrate conjugates, and amino acids or peptides (Figure 6 and Supplementary Information, Figure S1). Among amino acids, valine, leucine, isoleucine, alanine, threonine, ornithine, arginine, lysine, glutamine and N-acetyl glutamine, glutamate, methionine, acetyl-carnitine, proline, asparagine, glycine, tyrosine, phenylalanine, methylhistidine, and tryptophan were revealed (Supplementary Information, Figure S1). Carboxylic acids were represented by acetone, acetate, acetoacetate, succinate, citrate, aspartate, fumarate, and formate (Supplementary Information, Figure S1). Other metabolites such as sugars (α/β glucose), nucleosides and cofactors (NAD+, NADP+, UDP-N-Acetyl-glucosamine and ATP/ADP), and other osmolytes, such as taurine, trigonelline, scyllo-inositol, and choline moieties (phosphocholine and glycerophosphocholine), were also revealed (Figure 6, and Supplementary Information, Figure S1).

## 4. Discussion

The murine *B16-F0* cell line is commonly used in studies focusing on melanogenesis or skin injury exploration [4,25,29,32], for instance, to examine the effects of natural or synthetic compounds on melanin synthesis [25,33,34,35]. Melanin production might be modulated by synthetic or natural compounds such as extracts from some higher plants [36]. For instance, the natural flavonoid tangeretin generates melanin production in *B16-F0* murine melanoma cells in a concentration-dependent manner [37]. Also, extracts of the *Piper nigrum* fruit and pure piperine induce skin darkening and melanin dispersal responses in the frog *Rana tigerina* [37], while berberine, an active molecule of *Berberis vulgaris*, increases melanin in the toad *Bufo melanostictus* melanophores [38]. A recent in silico study indicates that naringenin retrieved from the mangrove tree *Rhizophora mucronata* can stimulate the melanogenic pathway [39].

Melanogenesis, resulting in the synthesis of melanin—a photoprotective and antioxidant pigment [40]—starts with tyrosine conversion into L-3,4-dihydroxyphenylalanine (L-DOPA), catalyzed by tyrosinase (TYR). L-DOPA is subsequently oxidized to DOPAquinone, producing either eumelanin (brown-black pigment) or pheomelanin (yellow-red pigment), depending on the presence of substrates and enzymes such as cysteine and tyrosinase-related proteins (TRP1, TRP2) [25]. The onset of melanogenesis can be mediated by the mitogen-activated protein kinases (MAPKs) ERK1/ERK2 phosphorylation, activating the Microphthalmia-Associated Transcription Factor (MITF) [40]. ERK2 plays a more significant role in the intracellular melanogenesis process, compared to ERK1, since its ERK2 activation specifically induces epithelial-to-mesenchymal transition (EMT) and increases levels of mesenchymal markers like N-cadherin and fibronectin [41].

While melanin protects against UV damage, the oxidative reactions involved in melanin synthesis generate ROS in melanocytes, potentially affecting cell health [42]. ROS accumulation may lead to DNA damage, lipid peroxidation, and increased production of pro-inflammatory and anti-melanogenic cytokines, contributing to the autoimmune pathogenesis of *vitiligo* [6]. This is a crucial aspect to cope with when looking for a melanogenesis inducer solution, the latter being required to be melanogenic and antioxidant.

To further highlight the comparative effects, it is noteworthy that the *Odontella aurita* methanolic extract demonstrates a broader and more sustained activation of the melanogenic pathway than both tangeretin and fucoidan. While tangeretin primarily induces ERK1 phosphorylation and targets TRP1 and TRP2 genes at later time points, *O. aurita* extract initiates TYR activation rapidly (after 0.5 h), followed by sequential upregulation of TRP1 and TRP2, thereby prolonging tyrosinase synthesis and activity. In contrast, fucoidan—a sulfated polysaccharide from brown algae—inhibits melanogenesis by downregulating tyrosinase [43,44].

Moreover, unlike tangeretin and fucoidan, which are single-compound or structurally homogeneous inducers, the *O. aurita* methanolic extract offers a unique synergistic effect due to its complex mixture of antioxidants, amino acid precursors, and metabolic cofactors. This multifaceted composition not only enhances pigmentation more efficiently but also provides robust protection against oxidative stress generated during melanin biosynthesis. Thus, *O. aurita* extract stands out by combining potent melanogenic induction with antioxidant defense, offering advantages over tangeretin and fucoidan in both efficacy and cellular protection.

The higher values of ABTS compared to ORAC or FRAP are ascribable to the different targets of these measurements of antioxidant activity and to the fact that ABTS is related to both lipophilic compounds (such as carotenoids) and lipophilic–hydrophilic phenolic compounds, resulting in a highly responsive antioxidant test [26].

Methanolic extract of *O. aurita* exerts its melanogenic effects through gene regulation, protein synthesis, increased enzymatic activity, and antioxidant activity. *O. aurita* outperforms tangeretin in promoting melanin production and supports faster melanin synthesis onset shortly after treatment on the *B16-F0* cell line and maintains higher and more stable tyrosinase activity. Overall, *O. aurita* extract fosters an intracellular environment conducive to melanin production while mitigating cellular damage. This dual functionality probably leads to the maintenance of melanogenic effect for at least 96 h and thus highlights the high potential of *O. aurita* extracts.

The diatom extract efficiently upregulates the tyrosinase gene and enzyme synthesis. Our results indicate that *O. aurita* extract induces in a greater extent the phosphorylation of ERK2 compared to ERK1, while tangeretin overexpression only mediates ERK1 phosphorylation, which leads to increased pigmentation and involvement of the MITF pathway [45].

Also, *O. aurita* extract triggers gene upregulation starting with TYR activation after 0.5 h of treatment, followed by TRP1 at 1 h and TRP2 at 8 h. This sustained gene upregulation supports prolonged tyrosinase production and thus activity. The TYR gene regulates melanin production by catalyzing the early rate-limiting reaction, while TRP1 and TRP2 are involved in subsequent steps [45]. Conversely, tangeretin targets the TRP1 (after 4 h) and TRP2 genes (at 0.25 and 2 h), but not TYR.

Another crucial aspect to cope with when looking for a melanogenesis inducer solution is that the latter needs to be melanogenic and antioxidant. In this frame, extracts are of more interest than pure molecules, thanks to the potential positive interaction between molecules and/or cellular targets [46]. Amino acids like tyrosine and phenylalanine serve as precursors in melanin biosynthesis [47]. Also, *O. aurita* has a high content of antioxidant molecules such as carotenoids, vitamins, and phenolic compounds that can scavenge ROS generated by tyrosinase activity [48]. Quaternary ammonium salts can influence melanin synthesis rates and efficiency by interacting with melanin precursors and modulating tyrosinase activity [47]. Their role, alongside other metabolic effectors and enzymatic cofactors, adds complexity to melanin synthesis regulation [45].

Taurine and choline derivatives stabilize cellular environments, promoting conditions that favor pigment production [49]. Cofactors such as NAD+ and ATP may enhance enzymatic activity and act as vital catalysts, boosting biochemical processes underlying melanogenesis [49].

Also, some carboxylic acids may affect tyrosinase activity positively, impacting melanin production. Carboxylic acids such as ascorbic acid (vitamin C, present in *O. aurita* methanolic extract), kojic acid derivatives, fumaric acid, and certain alpha-hydroxy acids have demonstrated the ability to modulate tyrosinase activity [50]. Additionally, azelaic acid has been observed at lower doses to stimulate melanogenic activity by increasing tyrosinase mRNA expression in melanocytes [51].

Taken together, these findings suggest that *O. aurita* extract accelerates the onset of melanin synthesis and sustains a heightened level of enzymatic activity over time, distinguishing its action from that of single-compound inducers such as tangeretin. By fostering a cellular environment enriched with metabolic cofactors, antioxidants, and pigment-precursor amino acids, the extract orchestrates a multifaceted response that enhances pigmentation while shielding cells from the collateral oxidative stress associated with melanin biosynthesis.

The present study demonstrates the potent melanogenic and antioxidant capabilities of the *Odontella aurita* methanolic extract, particularly in comparison with other known inducers such as tangeretin and fucoidan. The extract’s ability to upregulate key melanogenic genes and maintain robust tyrosinase activity suggests significant potential for therapeutic and cosmetic applications targeting pigmentation and cellular protection.

However, an important consideration for translational relevance is how the concentrations tested in vitro relate to levels that would be suitable or safe for human consumption. The concentrations used in the *B16-F0* cell line experiments are typically expressed in µg or ng per mg dry weight (DW) and are optimized for cell culture conditions to elicit measurable biological responses. In contrast, for human applications, both efficacy and safety must be considered, particularly in terms of systemic exposure, metabolic processing, and potential toxicity.

The tested concentrations—such as 0.78 (±0.06) ng mg DW^−1^ for carotenoids and 26.09 (±2.89) pmol mg DW^−1^ for Zn^2+^—are within the range commonly employed for in vitro studies to ensure cellular uptake and response. When extrapolating to human use, these values must be interpreted carefully. For topical applications, the effective concentration at the skin surface is likely to be much lower than the concentrations directly applied to cell cultures, owing to factors such as skin permeability, dilution, and metabolic degradation. For oral or systemic intake, safety assessments need to consider availability, potential accumulation, and known safe limits of the extract’s individual components, including trace metals and antioxidants.

Current regulatory frameworks, such as those set by the European Food Safety Authority (EFSA) and the UK Food Standards Agency, provide guidelines for maximum permissible levels of various bioactive compounds in foods and supplements. For example, zinc intake is generally considered safe up to 40 mg per day for adults, and the polyphenol and carotenoid content in *O. aurita* is well within the levels found in many commonly consumed fruits and vegetables. Nevertheless, rigorous toxicological studies, including acute and chronic exposure assessments, are necessary before recommending specific dosages for human use.

It is also noteworthy that the complex mixture found in the *O. aurita* extract—encompassing antioxidants, amino acid precursors, and metabolic cofactors—may offer synergistic benefits but also introduces variables in safety and efficacy profiles. For topical products, patch testing and clinical evaluations would be essential to rule out irritancy or sensitization, while for dietary supplements, careful titration and monitoring of intake would be prudent.

In summary, while the concentrations tested in vitro demonstrate promising biological activity, further research is required to determine the optimal and safe levels for human consumption. This includes detailed pharmacokinetic studies, assessment of individual and combined component safety, and formulation development to ensure effective delivery without exceeding safe exposure limits. Such investigations will be crucial for translating these findings into practical and safe human applications.

## 5. Conclusions

Our study highlights the significant role of the extract from the diatom *O. aurita* as an inducer of melanin synthesis. By leveraging the synergistic effects of its bioactive components, *O. aurita* demonstrates considerable potential to enhance the melanogenic pathway while simultaneously mitigating oxidative stress. This nuanced mechanism may underpin its therapeutic promise in addressing pigmentary disorders, supporting skin health, and paving the way for its integration into novel cosmeceutical or nutraceutical applications. Further research is needed to elucidate the action mechanism more comprehensively, facilitating the development of organ-targeted studies, in vivo investigations, and clinical trials. Given that microalgae serve as an eco-sustainable resource for compound and process production and considering that *O. aurita* is included in the authorized list of food supplements, additional research investment is justified. The extract of Odontella aurita shows strong potential for promoting melanin synthesis and combating oxidative stress, highlighting its promise for treating pigmentary disorders and supporting skin health. To fully harness these benefits, future research should focus on the following:

- Clarifying the molecular mechanisms involved through advanced genetic and protein studies.
- Validating efficacy and safety with animal models and clinical trials.
- Developing effective formulations for both topical and oral use, ensuring stability and bioavailability.
- Conducting comprehensive safety studies, particularly around long-term use and possible interactions.
- Exploring sustainable, large-scale production to support practical applications.

From a practical perspective, *O. aurita* extract could lead to new, natural cosmeceutical and nutraceutical products for skin health and antioxidant protection. Its use also aligns with the growing trend toward sustainable, plant-based solutions in the health and wellness industry.

## Figures and Tables

**Table 1:** Antibodies (brand Antibodies.com) used for protein expression analysis and performing Western blot.

Cat. No.	Target	Host	Clonality	Dilution
A13410	Anti-Tyrosinase	Rabbit	Polyclonal	1:1000
A250256	Anti-TRP1	Mouse	Monoclonal	1:200
A90631	Anti-TRP2	Rabbit	Polyclonal	1:2000
A8261	Anti-Erk1 + Erk2	Rabbit	Monoclonal	1:5000

**Table 2:** Antibodies used for vitamin determination and performing competitive ELISA assay.

Target	Code	Brand
Anti-Vitamin C	PAA913Ge01	Cloud Clone
Anti-Vitamin D_2_	PAA921Ge01	Cloud Clone
Anti-Vitamin D_3_	PAA920Ge01	Cloud Clone
Anti-Vitamin E	DPAB-DC3974	Creative Diagnostics

**Table 3:** Bioactive compound content and antioxidant activity of *O. aurita*. TCC = Total carotenoid content, DW = Dried biomass, TPC = Total phenolic content, GAE = Gallic acid equivalent, TFC = Total flavonoid content, QE = Quercetin equivalent, Zn^2+^ = Zinc ion, ABTS = 2,2′-azinobis(3-ethylbenzothiazoline-6-sulphonic acid), TE = Trolox equivalent, ORAC = Oxygen radical absorbance capacity, FRAP = Ferric reducing antioxidant power.

	Content
TCC	14.63 (±0.97) µg mg DW^−1^
Fucoxanthin	11.16 (±0.81) µg mg DW^−1^
Diadinoxanthin	2.14 (±0.15) µg mg DW^−1^
β-carotene	0.82 (±0.03) µg mg DW^−1^
Cis-Fucoxanthin	0.26 (±0.02) µg mg DW^−1^
Diatoxanthin	0.17 (±0.01) µg mg DW^−1^
Zeaxanthin	0.04 (±0.01) µg mg DW^−1^
Diadinochrome	0.02 (±0.002) µg mg DW^−1^
Violaxanthin	0.02 (±0.004) µg mg DW^−1^
TPC	18.11 (±1.53) GAE mg DW^−1^
TFC	1.56 (±0.07) QE mg DW^−1^
Vitamin C	0.27 (±0.014) µg mg DW^−1^
Vitamin D_2_	385.47 (±12.31) ng mg DW^−1^
Vitamin D_3_	15.33 (±0.72) ng mg DW^−1^
Vitamin E	0.78 (±0.06) ng mg DW^−1^
Zn^2+^	26.09 (±2.89) pmol mg DW^−1^
ABTS	7.45 (±0.08) µg TE mg DW^−1^
ORAC	0.33 (±0.03) µg TE mg DW^−1^
FRAP	0.13 (±0.002) µg TE mg DW^−1^

## Data Availability

Data available/the data that has been used is contained in this manuscript and Supplementary Information.

## Supplementary Materials

The following supporting information can be downloaded at https://www.mdpi.com/article/10.3390/antiox14121402/s1: Figure S1: Serial dilutions of melanin were prepared in phosphate-buffered saline, and their absorbance was measured at 405 nm using a microplate reader. The resulting standard curve enabled accurate interpolation of melanin concentrations in experimental samples by comparing their absorbance values to the established reference points. This method ensures reliable assessment of melanin production across treatments. Figure S2: Unprocessed original membranes of Western blotting. Figure S3: Typical 1H proton NMR 600 MHz profile of diatom *Odontella aurita* polar fraction. (A) High field and (B) low field spectrum showing main polar resonances and information. The *X*-axis represents the chemical shifts (ppm) of the peaks in the entire spectrum, and the *Y*-axis represents the intensity values. Table S1: Primer sequences for genes involved in melanogenesis; Metabolites assignments is reported in Table S2; Table S2: ^1^H and ^13^C chemical shift assignment (d, ppm) of metabolites found in ^1^H-TOCSY, ^1^H-^13^C-HSQC, and ^1^H-^13^C-HMBC -NMR spectra of *Odontella aurita* diatom hydrophilic extracts. Tables S3–S4. The tables present the results of a cell viability assay performed on B16 F0 and HeMa cells that were treated with three different agents: *Odontella aurita*, tangeretin, and fucoidan.

## Abbreviations

The following abbreviations are used in this manuscript:

ABTS	2,2′-azino-bis(3-ethylbenzothiazoline-6-sulfonic acid)
ACTB	β Actin
AlCl3	Aluminum chloride
BSA	Bovine Serum Albumin
DMEM/F-12	Dulbecco’s Modified Eagle Medium/Nutrient Mixture F-12
DMSO	Dimethyl sulfoxide
Dt	Diatoxanthin
DW	Dry weight
ELISA	Enzyme-linked immunosorbent assay
EMT	Epithelial–Mesenchymal Transition
ERK1	Extracellular Signal-Regulated Kinase 1
ERK2	Extracellular Signal-Regulated Kinase 2
EU	European Union
FBS	Fetal Bovine Serum
FRAP	Ferric ion reducing antioxidant power
GAE	Gallic Acid Equivalent
HEMa	Human epidermal melanocytes
HPLC	High pressure liquid chromatography
HRP	Horse Radish Peroxidase
HSQC	Heteronuclear single quantum coherence
L-DOPA	L-3,4-dihydroxyphenylalanine
LED	Light Emitting Diode
MITF	Microphthalmia associated transcription factor
MTT	3-(4,5-dimethylthiazol-2-yl)-2,5-diphenyltetrazolium bromide
Na_2_CO_3_	Sodium carbonate
NAD	Nicotinamide Adenine Dinucleotide
NaOH	Sodium Hydroxide
NMR	Metabolomics using nuclear magnetic resonance
NORCAA	The Norwegian Culture Collection of Algae
ORAC	Oxygen Radical Absorbance Capacity
PAR	Photosynthetically Active Radiation
PBS	Phosphate-Buffered Saline
PMSF	Phenylmethanesulfonyl fluoride
QE	Quercetin Equivalent
RGB	Red:Green:Blue
RIPA	Radio-Immunoprecipitation Assay
ROS	Reactive Oxygen Species
RT	Room Temperature
RT-qPCR	Reverse Transcription quantitative Polymerase Chain Reaction
SD	Standard Deviation
SDS-page	Sodium Dodecyl Sulfate Polyacrylamide Gel Electrophoresis
TBS	Tris Buffered Saline
TCA	Trichloroacetic acid
TCC	Total carotenoid content
TE	Trolox equivalent
TFC	Total flavonoid content
TOCSY	Clean total-correlation spectroscopy
TPC	Total phenolic content
TRP1	Tyrosinase-related protein 1
TRP2	Tyrosinase-related protein 2
TYR	Tyrosinase
UV	Ultraviolet
*v*/*v*	Volume/volume
*w*/*v*	Weight/volume
Zn^2+^	Zinc ion
ZnCl_2_	Zinc chloride
