# Supplementary material for: The Diatom Odontella aurita Modulates Melanogenesis in B16-F0 Cell Line
Source: Antioxidants (Basel). 2025 Nov 25;14(12):1402. doi: 10.3390/antiox14121402 (PMC12730007; doi:10.3390/antiox14121402)
Supplement: Supplementary file 1 [file antioxidants-14-01402-s001.zip › antioxidants-3935258-supplementary.pdf]

Table S1. Primer sequences for genes involved in melanogenesis

Gene	Primer	Sequence 5′=>3′	Length	Yield [nmol]	Volume for 100 pmol/μL [μL]	Tm [°C]	GC content [%]
**TYR**	TYR-F	CAGATCTCTGATGGCCAT	18	33.2	332	58.6	50
	TYR-R	GGATGACATAGACTGAGC	18	33.7	337	52.4	50
**TRP1**	TRP1-F	CTTTCTCCCTTCCTTACTGG	20	40.0	400	59.3	50
	TRP1-R	TGGCTTCATTCTTGGTGCTT	20	37.8	378	64.6	45
**TRP2**	TRP2-F	TGAGAAGAAACAAAGTAGGCA GAA	24	34.2	342	63.0	37.5
	TRP2-R	CAACCCCAAGAGCAAGACGAA AGC	24	52.4	524	72.3	54.2
**ACTB**	ATCB-F	ATGGGTCAGAAGGACTCCTAC G	18	42.9	429	65.4	66.6
	ACTB-R	AGTGGTA CGACCAGAGGCATAC	18	44.5	445	64.1	66.6

Note: TYR = Tyrosinase, TRP1 = Tyrosinase-Related Protein 1, TRP2 = Tyrosinase-Related Protein 2, ACTB = β-actin.

Table S2. ^{1}H and ^{13}C chemical shift assignment (d, ppm) of metabolites found in ^{1}H-TOCSY, ^{1}H-^{13}C-HSQC and ^{1}H-^{13}C-HMBC -NMR spectra of *Odontella aurita* diatom hydrophilic extracts.

**Metabolites**	**d ^{1}H**	**d ^{13}C**	**Group[a]**	**Metabolites**	**d ^{1}H**	**d ^{13}C**	**Group[a]**
Acetate	1.92	23.59	bCH_3	Methionine	2.13<br>2.15<br>2.65<br>3.89		S-CH_3<br>bCH_2<br>gCH_2<br>aCH
Acetoacetate	2.27	33.02	N-CH_3	Myo-Inositol	3.29<br>3.54<br>3.64<br>4.06	 73.11<br>71.60 	C5H<br>C3H<br>C4,6H<br>C2H
Acetone	2.24	24.05	bCH_2	N-Acetyl Gln*	1.93<br>2.04<br>2.34<br>4.20	 20.08<br>68.90 	 bCH_3<br>aCH
Acetyl Carnitine	5.61<br>3.20<br>2.65		bCH<br>N$(CH_3)_3$<br>gCH_2	NAD+*	9.33<br>9.13<br>8.84<br>8.19<br>6.04<br>6.09		N2 ring<br>N6 ring<br>N4 ring<br>N5 ring<br>N1'H<br>A1'H
a-glucose				NADP+*			

	5.24<br>3.54<br>3.72<br>3.42<br>3.84<br>3.78	92.40<br>72.20<br>73.30<br>70.03<br>71.61<br>62.37	C1H<br>C2H<br>C3H<br>C4H<br>C5H<br>C6H		9.30<br>9.09<br>8.84<br>8.15<br>6.06<br>6.10		N2 ring<br>N6 ring<br>N4 ring<br>A2H ring<br>N1'H<br>A1'H
Alanine	1.48<br>3.78	16.47<br>51.40	bCH$_3$<br>aCH	Ornithine	1.76<br>1.83<br>1.94<br>3.03<br>3.80	58.00	bCH$_3$<br>aCH$_2$
Arginine	1.65<br>1.91<br>3.25<br>3.78	24.30<br>28.04<br><br>54.80	gCH$_2$<br>bCH$_2$<br>gCH$_3$<br>aCH	Phenylalanine	7.34<br>7.37<br>7.43	129.02<br>138.03<br>127.00	C4 ring<br>C2,6 ring<br>C3,5 ring
Asparagine	2.86<br>2.96<br>4.01	36.90	bCH<br>b'CH<br>aCH	Phosphocholine	3.22<br>3.60<br>4.16	54.29	N-CH$_3$<br>CH$_2$<br>CH$_2$
Aspartate	2.69<br>2.82<br>3.91	37.10	bCH<br>b'CH<br>aCH	Proline	2.06<br>2.34<br>3.35<br>3.43<br>4.15	29.30<br>29.30<br>46.50<br><br>61.60	b′CH<br>bCH<br>d′CH<br>dCH<br>aCH
ATP*	8.26<br>8.54<br>6.15		C2 ring<br>NH ring<br>C1'H ribose	Propionate	1.05<br>2.19	17.30<br>58.00	bCH$_3$<br>aCH$_2$
b-glucose	4.64<br>3.26<br>3.48<br>3.40<br>3.47<br>3.90	96.40<br>74.60<br>76.22<br>70.70<br>76.80<br>61.40	C1H<br>C2H<br>C3H<br>C4H<br>C5H<br>C6H	Saturated Fatty Acids	0.89<br>1.29	12.06	ωCH3<br>CH2-CH3
Choline	3.20<br>3.51<br>4.07	54.38	N-CH$_3$<br>CH$_2$<br>CH$_2$	Scyllo-Inositol	3.35	74.20	CH$_2$

Formate	8.47	171.50	HCOO-	Succinate	2.41	34.30	a,bCH$_2$
Fumarate	6.52	136.30	a,bC=C	Taurine	3.27<br>3.43	54.47<br>66.50	N-CH$_3$<br>aCH$_2$
Glutamate	2.09<br>2.35<br>3.76	27.60<br>33.90<br>55.12	bCH<br>gCH$_2$<br>aCH	Threonine	1.33<br>3.59<br>4.27	17.30<br>58.00	gCH$_3$<br>aCH<br>bCH
Glutamine	2.17<br>2.42<br>3.77	27.55<br><br>55.12	bCH$_2$<br>gCH$_2$<br>aCH	t-methylhistidine	7.06<br>7.65		C4H ring<br>C2H ring
Glycero-phosphocholine	3.23<br>3.62<br>4.33	54.42	N-CH$_3$<br>N-CH$_2$<br>CH$_2$	Trigonelline	8.09<br>8.84<br>9.13		C4 ring<br>C3,5 ring<br>C1 ring
Glycine	3.57	42.00	aCH	Tryptophan	7.74<br>7.54<br>7.30<br>7.21	118.90<br>112.70<br>126.20<br>120.00	C4H ring<br>C7H ring<br>C6H ring<br>C5H ring
Isoleucine	0.93<br>1.01<br>1.29<br>1.47<br>1.98	11.29<br>15.23	dCH$_3$<br>gCH$_3$<br>gCH<br>g'CH<br>bCH	Tyrosine	6.90<br>7.20	116.30<br>131.10	C3,5H ring<br>C2,6H ring

Leucine	0.95	22.29	dCH_3	UDPN-Acetyl Gln*	7.95		C1H ribose
	0.97	21.97	gCH_3		5.97		C1H ribose
	1.72	40.30	gCH		5.52		C6 ring
	3.72	61.81	bCH_2				
Lysine	1.46	21.99	gCH_2	Valine	0.99	17.32	gCH_3
	1.73	26.80	dCH_3		1.04	18.35	$g'CH_3$
	1.91	30.29	bCH_2		2.28	29.90	bCH
	3.03	39.70	eCH_2 aCH		3.62		aCH
	3.74	54.80					
Methanol	3.36	49.90	CH_3				

*Abbreviations: N-Acetyl Gln = N-acetyl-glutamine; ATP = Adenosine triphosphate; NAD+ = nicotinamide adenine dinucleotide oxidized; NADP = Nicotinamide adenine dinucleotide phosphate oxidized; UDPN-Acetyl Gln = Uridine diphosphate-N-Acetyl-D-Glucosamine.

[a]A = adenine ring; A' = ribose moiety of adenine nucleotide group; N = nicotinamide ring; N' = ribose moiety of nicotinamide nucleotide group.

Table S3. The table presents the results of a cell viability assay performed on B16 F0 cells that were treated with three different agents: Odontella aurita, tangeretin, and fucoidan. The first column represents untreated cells, which serve as the control group. For each treatment, the table displays the percentage of viable cells (% cell viability), the standard deviation (SD±), and the concentration of the compound tested (either in μg/mL or μM, as appropriate). For Odontella aurita, the tested concentrations range from 0 to 100 μg/mL. Cell viability remains high across all tested concentrations, with slight variations and standard deviations typically between 1 and 5. Tangeretin is tested across concentrations from 0 to 100 μM. At lower concentrations, cell viability is close to that of the control, but as the concentration increases (notably at 10 μM and above), a marked decrease in viability is observed, with the lowest value being 41% at 50 μM. Fucoidan is tested at concentrations up to 100 μg/mL, and the cell viability remains relatively high and stable, similar to the control group, across all tested concentrations. Overall, the data suggest that Odontella aurita and fucoidan have minimal cytotoxic effects on B16 F0 cells at the concentrations tested, while tangeretin shows a significant reduction in cell viability at higher concentrations.

**Untreated**		***Odontella aurita***			**Tangeretin**			**Fucoidan**		
**% cell viability**	**SD±**	**Concentration tested $\mu g\ mL^{-1}$**	**% cell viability**	**SD±**	**Concentration tested μM**	**% cell viability**	**SD±**	**Concentration tested $\mu g\ mL^{-1}$**	**% cell viability**	**SD±**
100	5	0	99	2	0	99	2.3	0	99	2
99	3	0.1	95	1.3	6	97	5.1	0,1	96	11.0
99	2	0.5	99	8.3	13	89	3.6	10	98	2.7
98	5	0.75	97	12.7	25	67	7.1	100	96	1.9
97	1	1	99	4.8	50	41	3.5			
96	2	1.25	98	4.4						
99	3	1.5	99	1.6						
100	2	3	97	1.8						
100	1	5	97	1.9						
100	2	7.5	96	0.9						
100	3	10	96	2.7						
95	2	25	94	2.8						
96	1	50	99	0.4						
98	2	75	92	1.8						
99	2	100	99	0.7						

Table S4. Effects of *Odontella aurita* extract, Tangeretin, and Fucoidan on cell viability of normal human melanocytes (HEMA cells) at various concentrations. Data are presented as percentages of cell viability (mean ± SD) for each compound and concentration tested. Odontella aurita extract maintains high cell viability (84–96%) across all doses, indicating minimal cytotoxicity. In contrast, tangeretin and fucoidan induce a concentration-dependent decrease in cell viability, with tangeretin showing a more pronounced effect at higher concentrations.

**Untreated**		***Odontella aurita***			**Tangeretin**			**Fucoidan**		
**% cell viability**	**SD±**	**Concentration tested μg mL^{-1}**	**% cell viability**	**SD±**	**Concentration tested μM**	**% cell viability**	**SD±**	**Concentration tested μg mL^{-1}**	**% cell viability**	**SD±**
87	5	0	100	0	0	99	2.3	0	84	6,0
89	3	0.1	86	3.5	6	87	0.4	0,1	65	0
86	2	0.5	82	0.7	13	88	6.5	10	76	0,2
87	5	0.75	71	4.4	25	78	3.1	100	84	5,8
88	1	1	86	0.5	50	59	6.1			
96	2	1.25	76	0.1						
88	3	1.5	81	2.7						
85	2	3	83	3.7						
87	1	5	78	1.3						
87	2	7.5	86	5.6						
85	3	10	88	1.6						
84	2	25	73	2.4						
85	1	50	88	4.7						
88	2	75	66	8.2						
86	2	100	62	3.2						

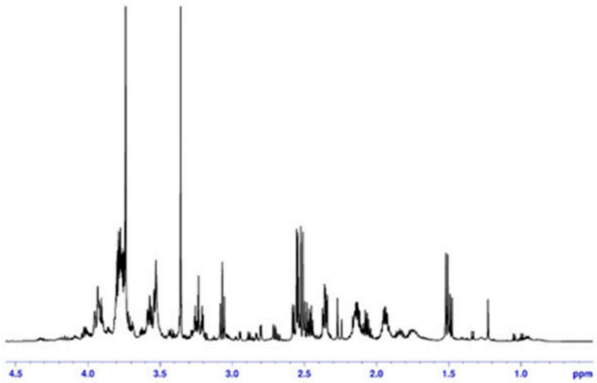

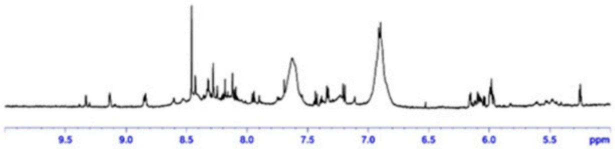

Figure S1. Serial dilutions of melanin were prepared in phosphate-buffered saline, and their absorbance was measured at 405 nm using a microplate reader. The resulting standard curve enabled accurate interpolation of melanin concentrations in experimental samples by comparing their absorbance values to the established reference points. This method ensures reliable assessment of melanin production across treatments.

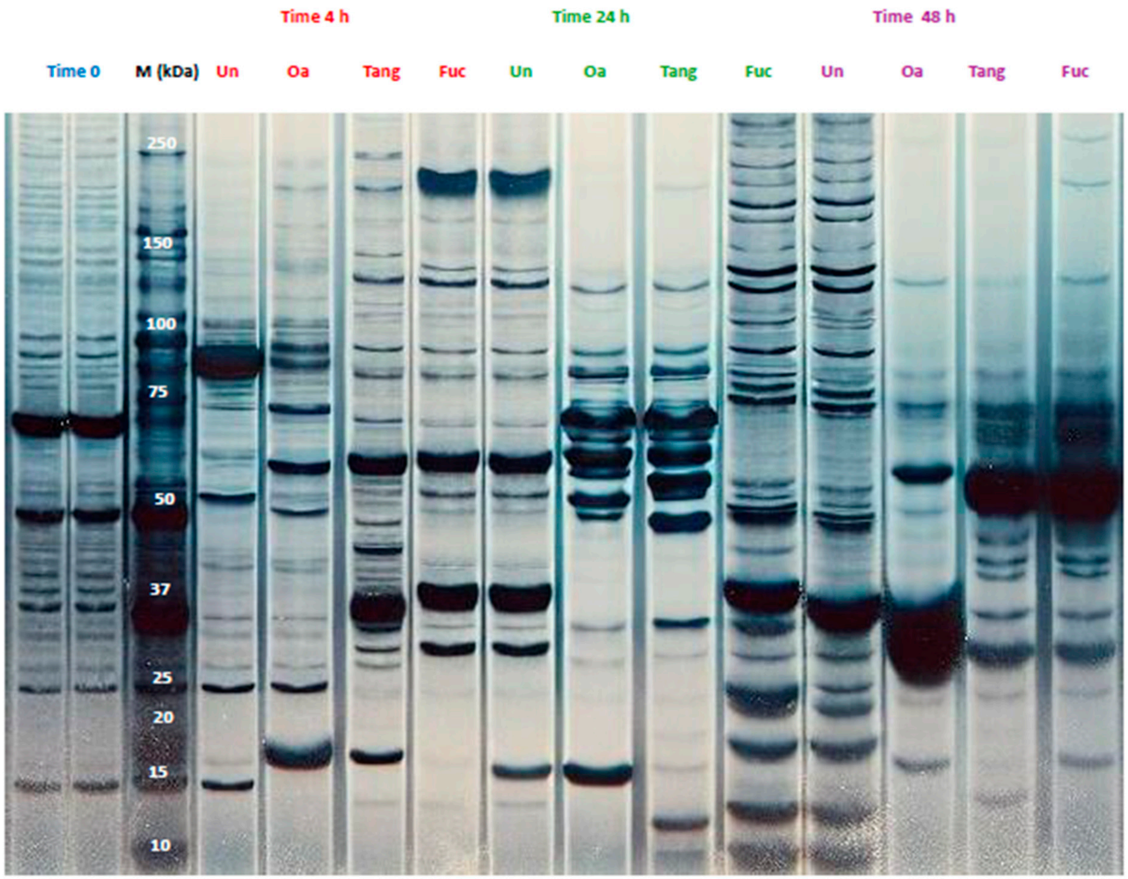

Figure S2. Unprocessed original membranes of Western blotting.

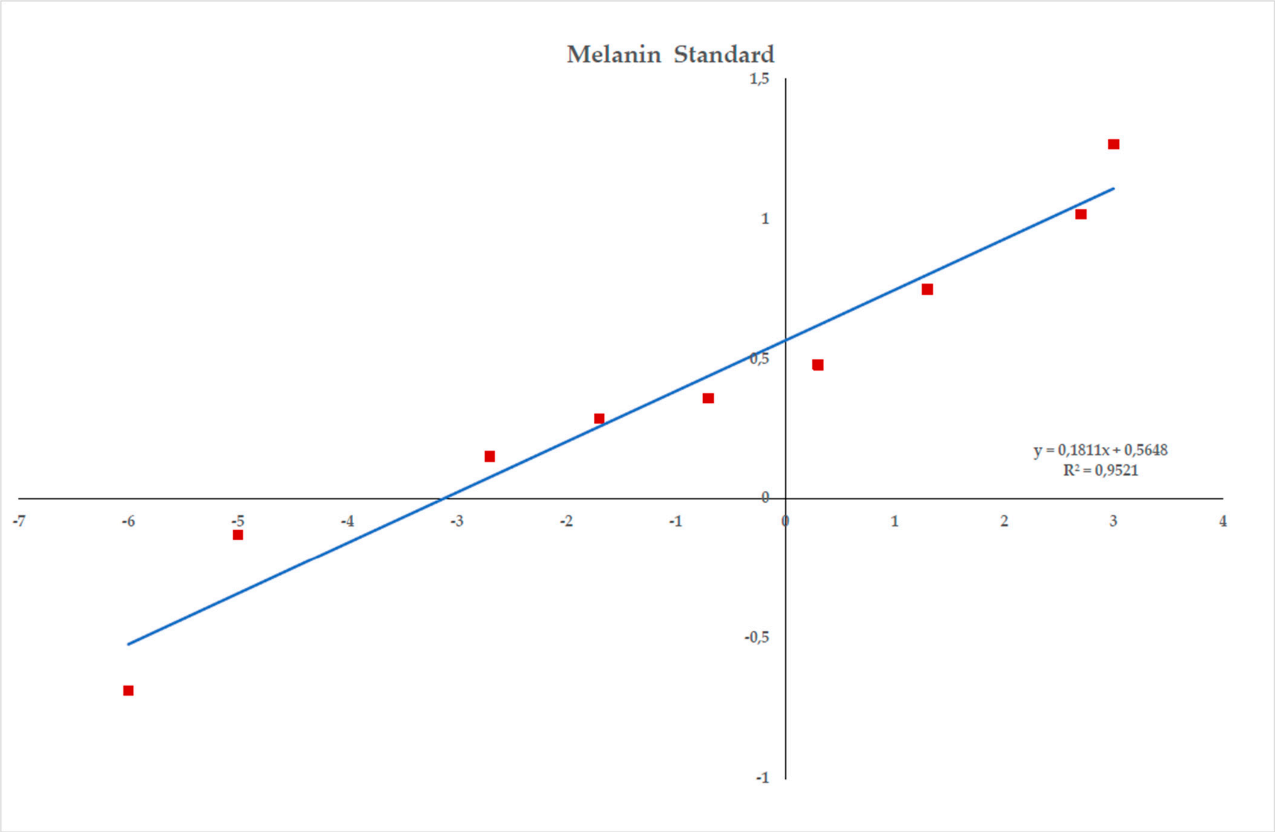

Figure S3. Typical 1H proton NMR 600 MHz profile of diatom *Odontella aurita* polar fraction. (A) High field and (B) low field spectrum showing main polar resonances and information. The *X*-axis represents the chemical shifts (ppm) of the peaks in the entire spectrum, and the *Y*-axis represents the intensity values.
